# Piperazine-1,4-diium bis­[tetra­chlorido­aurate(III)] dihydrate

**DOI:** 10.1107/S1600536809041063

**Published:** 2009-10-17

**Authors:** Anna V. Polishchuk, Emilia T. Karaseva, Mikhail A. Pushilin

**Affiliations:** a Institute of Chemistry FEB RAS, 159 Prospekt Stoletiya, Vladivostok 690022, Russian Federation

## Abstract

In the title compound, (C_4_H_12_N_2_)[AuCl_4_]_2_·2H_2_O, the Au^III^ atom has a square-planar geometry. The piperazinium dication lies on an inversion centre and adopts a typical chair conformation. In the crystal, a combination of N—H⋯O, N—H⋯Cl and O—H⋯Cl hydrogen bonds results in the formation of a three-dimensional network.

## Related literature

For bond distances, see: Allen *et al.* (1987 ▶). For similar compounds, see: Kefi & Nasr (2005 ▶); Sharutin *et al.* (2008 ▶); Sutherland & Harrison (2009 ▶); Zhang *et al.* (2006 ▶).
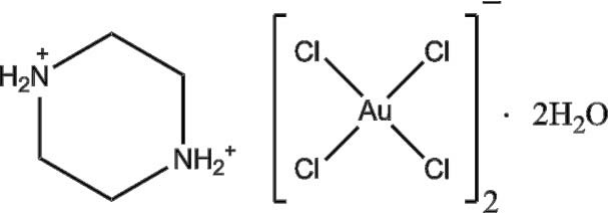


## Experimental

### 

#### Crystal data

(C_4_H_12_N_2_)[AuCl_4_]_2_·2H_2_O
*M* *_r_* = 801.72
Monoclinic, 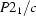

*a* = 7.7327 (11) Å
*b* = 10.1114 (15) Å
*c* = 11.9024 (18) Å
β = 105.565 (3)°
*V* = 896.5 (2) Å^3^
*Z* = 2
Mo *K*α radiation
μ = 17.53 mm^−1^
*T* = 296 K
0.33 × 0.23 × 0.08 mm

#### Data collection

Bruker SMART CCD 1000 diffractometer
Absorption correction: gaussian (*XPREP* and *SADABS*; Bruker, 2003 ▶) *T*_min_ = 0.043, *T*_max_ = 0.251
6689 measured reflections
2630 independent reflections
2446 reflections with *I* > 2σ(*I*)
*R*_int_ = 0.018

#### Refinement

*R*[*F* ^2^ > 2σ(*F* ^2^)] = 0.018
*wR*(*F* ^2^) = 0.044
*S* = 1.09
2630 reflections
89 parameters
2 restraints
H atoms treated by a mixture of independent and constrained refinement
Δρ_max_ = 1.36 e Å^−3^
Δρ_min_ = −0.75 e Å^−3^

### 

Data collection: *SMART* (Bruker, 2007 ▶); cell refinement: *SAINT* (Bruker, 2007 ▶); data reduction: *SAINT*; program(s) used to solve structure: *SHELXS97* (Sheldrick, 2008 ▶); program(s) used to refine structure: *SHELXL97* (Sheldrick, 2008 ▶); molecular graphics: *SHELXTL* (Sheldrick, 2008 ▶); software used to prepare material for publication: *publCIF* (Westrip, 2009 ▶).

## Supplementary Material

Crystal structure: contains datablocks I, global. DOI: 10.1107/S1600536809041063/su2149sup1.cif

Structure factors: contains datablocks I. DOI: 10.1107/S1600536809041063/su2149Isup2.hkl

Additional supplementary materials:  crystallographic information; 3D view; checkCIF report

## Figures and Tables

**Table 1:** Hydrogen-bond geometry (Å, °)

*D*—H⋯*A*	*D*—H	H⋯*A*	*D*⋯*A*	*D*—H⋯*A*
N1—H1*B*⋯O1^i^	0.90	1.97	2.815 (3)	155
N1—H1*B*⋯O1^ii^	0.90	2.39	2.960 (3)	121
O1—H2⋯Cl1^iii^	0.839 (13)	2.57 (2)	3.3035 (19)	147 (3)
O1—H2⋯Cl4^iii^	0.839 (13)	2.83 (3)	3.445 (2)	131 (3)
O1—H1⋯Cl4	0.822 (14)	2.71 (3)	3.382 (2)	140 (3)
O1—H1⋯Cl3	0.822 (14)	2.67 (3)	3.268 (2)	131 (3)
N1—H1*A*⋯Cl1	0.90	2.60	3.373 (2)	144
N1—H1*A*⋯Cl2	0.90	2.81	3.575 (2)	143

Symmetry codes: (i) 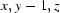; (ii) 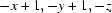; (iii) 
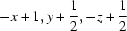
.

**Fig. 1.**
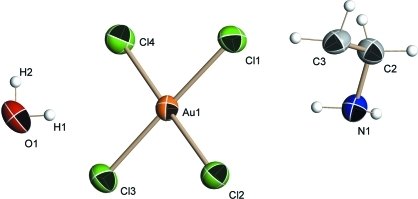
The asymmetric unit of the title compound, showing 50% displacement ellipsoids (arbitrary spheres for the H atoms).

**Fig. 2.**
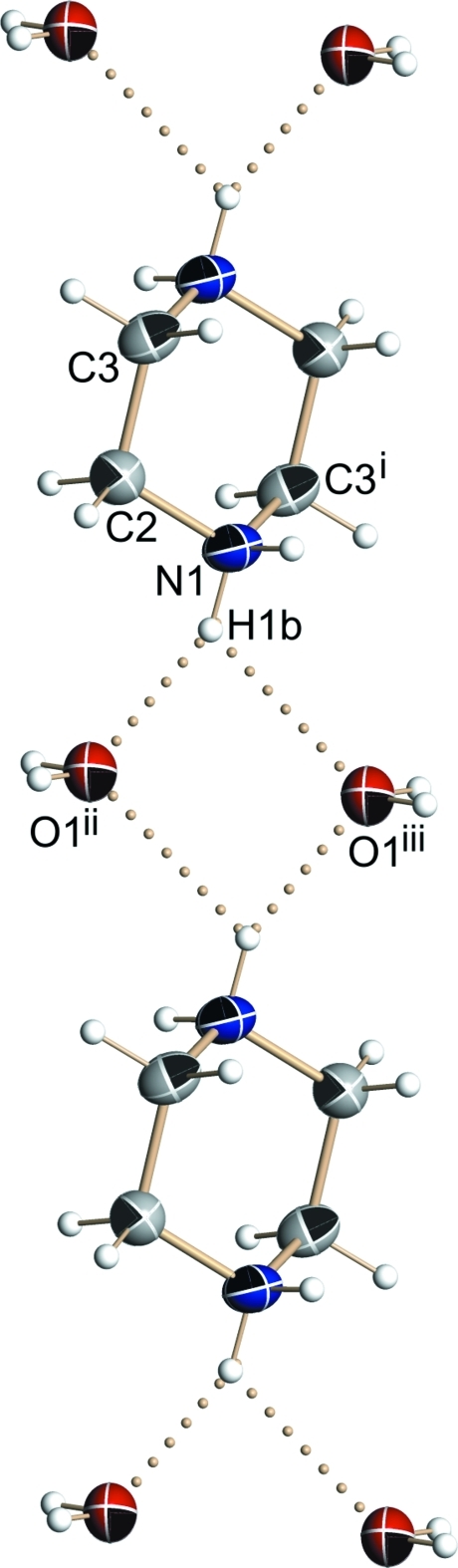
Fragment of the water-piperazinium hydrogen bonded chain, with the hydrogen bonds indicated by dotted lines. Symmetry codes are the same as in Table 1.

**Fig. 3.**
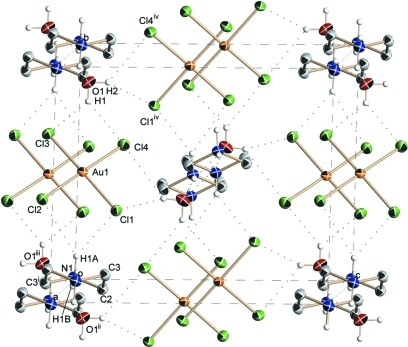
A view along the a axis of the crystal packing of the title compound, with the hydrogen bonds shown as dotted lines. All the C-bound H atoms have been omitted for clarity. Symmetry codes are the same as in Table 1.

## supplementary crystallographic information

### Comment

The asymmetric unit of the title compound consists of a discrete [AuCl_4_]^-^ complex anion, one water molecule and one-half of a diprotonated piperazinium dication (Fig. 1). The Au atom in the tetrachloridoaurate anion exhibits a square-planar coordination. A similar geometry has been observed, for exemple, in tetraphenylantimony(V) tetrachloroaurate (Sharutin *et al.*, 2008) and bipyridinium tetrachloroaurate (Zhang *et al.*, 2006). The Au—Cl bond lengths are in the range of 2.2802 (6) - 2.2842 (7) Å. In the crystal structure, the anions are stacked into columns along the *a *axis, parallel to each other. The distances between anion planes are ca. 3.734 and 3.999 Å. The organic piperazinium dication lies at an inversion centre and adopts a typical chair geometry with normal valence bond lengths (Allen *et al.*, 1987) and angles, as observed in the structures of piperazinediium tetrachloridozincate (Sutherland & Harrison, 2009) and piperazinediium tetrachloridozincate monohydrate (Kefi & Nasr, 2005).

The piperazinium dications and water molecules are linked by intermolecular bifurcated N—H···O hydrogen bonds to form chains proagagting along the [100] direction (Fig. 2). The water-piperaziniun chains and the anion stacks form a three-dimensional framework (Fig. 3) via bifurcated N—H···Cl and O—H···Cl hydrogen bonds (Table 1).

### Experimental

The chemicals used were of reagent grade. Ciprofloxacin hydrochloride (37 mg, 0.1 mmol) and gold(III) chloride (AuCl_3_ 30 mg, 0.1 mmol) were dissolved in 10 ml of 32% of HCl. Yellow crystals of the title compound, suitable for X-ray analysis, were obtained by slow evaporation in air at rt, after a few days.

### Refinement

The water H-atoms were located from difference electron-density maps and were refined with distance restraints of O—H = 0.85 (2) Å and U*iso*(H) = 1.5U*eq*(O). All the other H-atoms were positioned geometrically and allowed to ride on their parent atoms: N—H = 0.90 Å, C—H = 0.97 Å with U*iso*(H)= 1.2U*eq*(parent N or C atom).

### Crystal data

**Table:**

(C_4_H_12_N_2_)[AuCl_4_]_2_·2H_2_O	*F*(000) = 728
*M**_r_* = 801.72	*D*_x_ = 2.970 Mg m^−^^3^
Monoclinic, *P*2_1_/*c*	Mo *K*α radiation, λ = 0.71073 Å
Hall symbol: -P 2ybc	Cell parameters from 1008 reflections
*a* = 7.7327 (11) Å	θ = 3.4–30.6°
*b* = 10.1114 (15) Å	µ = 17.53 mm^−^^1^
*c* = 11.9024 (18) Å	*T* = 296 K
β = 105.565 (3)°	Prism, yellow
*V* = 896.5 (2) Å^3^	0.33 × 0.23 × 0.08 mm
*Z* = 2	

### Data collection

**Table:**

Bruker SMART CCD 1000 diffractometer	2630 independent reflections
Radiation source: fine-focus sealed tube	2446 reflections with *I* > 2σ(*I*)
graphite	*R*_int_ = 0.018
Detector resolution: 8.33 pixels mm^-1^	θ_max_ = 31.5°, θ_min_ = 2.7°
ω scans	*h* = −10→9
Absorption correction: gaussian (*XPREP* and *SADABS*; Bruker, 2003)	*k* = −13→12
*T*_min_ = 0.043, *T*_max_ = 0.251	*l* = −14→17
6689 measured reflections	

### Refinement

**Table:**

Refinement on *F*^2^	Secondary atom site location: difference Fourier map
Least-squares matrix: full	Hydrogen site location: inferred from neighbouring sites
*R*[*F*^2^ > 2σ(*F*^2^)] = 0.018	H atoms treated by a mixture of independent and constrained refinement
*wR*(*F*^2^) = 0.044	*w* = 1/[σ^2^(*F*_o_^2^) + (0.0203*P*)^2^ + 0.7643*P*] where *P* = (*F*_o_^2^ + 2*F*_c_^2^)/3
*S* = 1.09	(Δ/σ)_max_ = 0.002
2630 reflections	Δρ_max_ = 1.36 e Å^−^^3^
89 parameters	Δρ_min_ = −0.75 e Å^−^^3^
2 restraints	Extinction correction: *SHELXL97* (Sheldrick, 2008), Fc^*^=kFc[1+0.001xFc^2^λ^3^/sin(2θ)]^-1/4^
Primary atom site location: structure-invariant direct methods	Extinction coefficient: 0.01512 (17)

### Special details

**Table:**

Geometry. All e.s.d.'s (except the e.s.d. in the dihedral angle between two l.s. planes) are estimated using the full covariance matrix. The cell e.s.d.'s are taken into account individually in the estimation of e.s.d.'s in distances, angles and torsion angles; correlations between e.s.d.'s in cell parameters are only used when they are defined by crystal symmetry. An approximate (isotropic) treatment of cell e.s.d.'s is used for estimating e.s.d.'s involving l.s. planes.
Refinement. Refinement of *F*^2^ against ALL reflections. The weighted *R*-factor *wR* and goodness of fit *S* are based on *F*^2^, conventional *R*-factors *R* are based on *F*, with *F* set to zero for negative *F*^2^. The threshold expression of *F*^2^ > σ(*F*^2^) is used only for calculating *R*-factors(gt) *etc*. and is not relevant to the choice of reflections for refinement. *R*-factors based on *F*^2^ are statistically about twice as large as those based on *F*, and *R*- factors based on ALL data will be even larger.

### Fractional atomic coordinates and isotropic or equivalent isotropic displacement parameters (Å^2^)

**Table:**

	*x*	*y*	*z*	*U*_iso_*/*U*_eq_	
Au1	0.258077 (10)	0.481141 (8)	0.040268 (7)	0.03248 (2)	
Cl1	0.22668 (9)	0.31457 (6)	0.16243 (5)	0.04886 (14)	
Cl2	0.12348 (9)	0.34990 (6)	−0.11438 (5)	0.04885 (15)	
Cl3	0.29217 (10)	0.64504 (6)	−0.08394 (6)	0.05264 (16)	
Cl4	0.39054 (10)	0.60981 (7)	0.19756 (6)	0.05297 (16)	
O1	0.5108 (2)	0.88550 (19)	0.07637 (17)	0.0495 (4)	
H1	0.499 (5)	0.8047 (14)	0.075 (3)	0.074*	
H2	0.546 (5)	0.890 (4)	0.1494 (12)	0.074*	
N1	0.1859 (2)	0.02747 (18)	0.01336 (18)	0.0360 (4)	
H1A	0.1882	0.1163	0.0188	0.043*	
H1B	0.2985	−0.0004	0.0190	0.043*	
C2	0.1246 (3)	−0.0285 (2)	0.1115 (2)	0.0390 (5)	
H2A	0.2018	0.0029	0.1849	0.047*	
H2B	0.1330	−0.1242	0.1106	0.047*	
C3	−0.0679 (3)	0.0117 (2)	0.1018 (2)	0.0384 (5)	
H3A	−0.1087	−0.0305	0.1632	0.046*	
H3B	−0.0741	0.1067	0.1113	0.046*	

### Atomic displacement parameters (Å^2^)

**Table:**

	*U*^11^	*U*^22^	*U*^33^	*U*^12^	*U*^13^	*U*^23^
Au1	0.03239 (3)	0.02880 (4)	0.03751 (4)	0.00020 (3)	0.01157 (3)	−0.00039 (3)
Cl1	0.0685 (3)	0.0368 (3)	0.0414 (3)	−0.0146 (2)	0.0150 (2)	0.0013 (2)
Cl2	0.0623 (3)	0.0419 (3)	0.0398 (3)	−0.0061 (3)	0.0092 (2)	−0.0053 (2)
Cl3	0.0701 (4)	0.0397 (3)	0.0488 (3)	−0.0048 (3)	0.0172 (3)	0.0087 (2)
Cl4	0.0677 (3)	0.0412 (3)	0.0463 (3)	−0.0155 (3)	0.0088 (3)	−0.0056 (2)
O1	0.0402 (7)	0.0474 (9)	0.0594 (10)	0.0011 (7)	0.0107 (7)	0.0196 (8)
N1	0.0297 (7)	0.0361 (9)	0.0450 (9)	−0.0024 (6)	0.0151 (7)	−0.0022 (7)
C2	0.0357 (9)	0.0425 (12)	0.0386 (11)	0.0013 (8)	0.0096 (8)	0.0047 (8)
C3	0.0371 (9)	0.0442 (11)	0.0382 (10)	−0.0045 (8)	0.0176 (8)	−0.0048 (8)

### Geometric parameters (Å, °)

**Table:**

Au1—Cl1	2.2802 (6)	N1—H1A	0.9000
Au1—Cl2	2.2813 (6)	N1—H1B	0.9000
Au1—Cl3	2.2827 (7)	C2—C3	1.517 (3)
Au1—Cl4	2.2842 (7)	C2—H2A	0.9700
O1—H1	0.822 (14)	C2—H2B	0.9700
O1—H2	0.839 (13)	C3—H3A	0.9700
N1—C3^i^	1.482 (3)	C3—H3B	0.9700
N1—C2	1.486 (3)		
			
Cl1—Au1—Cl2	88.92 (3)	N1—C2—C3	110.55 (18)
Cl1—Au1—Cl3	178.87 (3)	N1—C2—H2A	109.5
Cl2—Au1—Cl3	90.39 (3)	C3—C2—H2A	109.5
Cl1—Au1—Cl4	89.95 (3)	N1—C2—H2B	109.5
Cl2—Au1—Cl4	178.82 (2)	C3—C2—H2B	109.5
Cl3—Au1—Cl4	90.74 (3)	H2A—C2—H2B	108.1
H1—O1—H2	94 (3)	N1^i^—C3—C2	110.31 (19)
C3^i^—N1—C2	112.20 (17)	N1^i^—C3—H3A	109.6
C3^i^—N1—H1A	109.2	C2—C3—H3A	109.6
C2—N1—H1A	109.2	N1^i^—C3—H3B	109.6
C3^i^—N1—H1B	109.2	C2—C3—H3B	109.6
C2—N1—H1B	109.2	H3A—C3—H3B	108.1
H1A—N1—H1B	107.9		

Symmetry codes: (i) −*x*, −*y*, −*z*.

### Hydrogen-bond geometry (Å, °)

**Table:**

*D*—H···*A*	*D*—H	H···*A*	*D*···*A*	*D*—H···*A*
N1—H1B···O1^ii^	0.90	1.97	2.815 (3)	155
N1—H1B···O1^iii^	0.90	2.39	2.960 (3)	121
O1—H2···Cl1^iv^	0.84 (1)	2.57 (2)	3.3035 (19)	147 (3)
O1—H2···Cl4^iv^	0.84 (1)	2.83 (3)	3.445 (2)	131 (3)
O1—H1···Cl4	0.82 (1)	2.71 (3)	3.382 (2)	140 (3)
O1—H1···Cl3	0.82 (1)	2.67 (3)	3.268 (2)	131 (3)
N1—H1A···Cl1	0.90	2.60	3.373 (2)	144
N1—H1A···Cl2	0.90	2.81	3.575 (2)	143

Symmetry codes: (ii) *x*, *y*−1, *z*; (iii) −*x*+1, −*y*+1, −*z*; (iv) −*x*+1, *y*+1/2, −*z*+1/2.
